# Factors associated with willingness to receive a novel community care service for older people in Foshan, China: a cross-sectional study

**DOI:** 10.1186/s12913-022-08004-3

**Published:** 2022-04-29

**Authors:** Fengjiao Xie, Aiwen Deng, Jianhao Chen, Ribo Xiong

**Affiliations:** 1 Department of general surgery, The Third Affiliated Hospital of Southern Medical University, Guangzhou, China; 2 Department of rehabilitation, The Seventh Affiliated Hospital, Southern Medical University, Foshan, China

**Keywords:** Community care, Older people, Community rehabilitation, Utilization, Rural, China

## Abstract

**Background:**

In China, the majority of community care for older people is planned and offered in a few large cities. The provision of community care to the rural population is a new concept. This study aimed to assess the prevalence of willingness to receive a novel community care service that incorporates community rehabilitation among older people, and identify the possible factors influencing utilization willingness in Foshan, China.

**Methods:**

A cross-sectional study was conducted involving 413 older people in Lishui county, China from January to March 2021. Trained staff interviewed older people using structured questionnaires. Multivariate logistic regression analysis was used to determine factors that were significantly associated with the willingness to receive this novel community care.

**Results:**

The prevalence of willingness to receive community care for older people was 61.9% (*n* = 245) in Foshan, China. Older people who were living alone or living with spouses were about two times significantly more likely to receive community care compared to those living with children/others (OR = 2.19, 95%CI:2.44 ~ 3.19, OR = 2.015, 95%CI: 1.39 ~ 2.23, respectively). Older people who lived closer to the community care center were about two times significantly more likely to receive community care compared to those who lived far away (OR = 2.10, 95%CI: 1.61 ~ 2.41). Older people with activity of daily living disability were about three times significantly more likely to receive community care compared to those without a disability (OR = 3.13, 95%CI: 2.38 ~ 3.29).

**Conclusions:**

A majority of rural older people were willing to receive this novel community care service that includes community rehabilitation. The findings suggest the need for policies and programs to not only improve the provision of this novel community care service but also promote its uptake among older people in the study setting.

## Background

Population aging has created an unprecedented global challenge. In China, the number of older people aged 60 and over reached 176 million, accounting for 13% of the Chinese population in 2019 [1]. The older people are a vulnerable group due to their complex and continuously changing health and social problems, all of which would increase the demand for a wide range of services over a long period of time [2].

Long-term care practices for older people in China have two modes, family support and institutional care [3]. However, both family support and institutional care have disadvantages and are not necessarily feasible options for older people. The first is the huge shortfall of family caregivers (often adult children) as younger people have increasingly moved away from home for work [4]. Thus, the functioning of home-based care has been weakened, and the availability of care by their offspring has become uncertain. The phenomenon is more evident in rural China with the increasing migration of young labor force to urban regions for better opportunities and income [5]. Meanwhile, institutional care is not accessible to a majority of older people because they are either too expensive or limited to older people with no children, no income, and no relatives [3].

Community care has emerged as a mode in which older people living at home and the community provides day care in the form of visiting service or staying in a daycare center. Under this model, older people do not need to move out of the home and could get support from family members at times [6]. Research has shown the effectiveness of this model in supporting older people to live independently within the community [ [7]]. However, knowledge about the extent of willingness to receive community care is limited. Available data regarding the proportion of older people who are willing to receive this community care service is varied across regions [8–10]. How the specific context affects the utilization willingness of community care and its influential factors continuously attract researcher’s interest.

Recent studies suggest that certain socioeconomic and demographic factors may be associated with willingness for long-term care among older people [8–10]. For example, older people who were living alone are more likely to receive community care [ [8]], while participants with higher educational levels and socioeconomic status were more likely to choose institutional care [9]. Other research has shown that older people tend to prefer institutional care when there is lower life satisfaction and family harmony [10]. The existing literature mainly focused on determinants of willingness for institutional care which has disparities in arrangements of organization, financing and delivering of care service from community care. Report on utilization willingness of community care is scant and inconclusive. Moreover, in their studies various types of community care have been identified with some having medical and health service and some merely providing basic domestic help. How the services can best be organized and coordinated, their effects on willingness to receive community care remains unclear. This is a limitation given the integrated development and pluralist provisions of community care in rural China in the past couple of years [3].

In China, the majority of community care service for older people is planned and offered in a few large cities. The provision of community care service to the rural population is a new concept [3]. Lishui county, where this study was conducted, was one of the pilot rural regions where free community care service that incorporates community rehabilitation was provided. There is however, limited understanding of how willing older people in Lishui county would use this service. Furthermore, the factors that may be associated with willingness to receive this service is not well-understood. Hence, this study aimed to assess the prevalence of willingness to receive a novel community care service among older people, and identify the possible factors influencing utilization willingness in Foshan, China.

## Methods

### Study setting

The study was carried out in Lishui county, Foshan, China. The county is located in the middle of Pearl River Delta, one of the most affluent rural regions in China. At the end of 2019, the population of Lishui county was 580,000, and the proportion of those aged over 60 reached 22.3% of the total population, higher than the national average of 13.0% [1].

Concurrently with the policy of long-term care reform in China, Lishui county wanted to develop a novel community care service for older people. Under this model, the county government is responsible for infrastructure construction and purchasing of service including daytime caring, domestic service, entertainment activities. The specific characteristic of this model is community rehabilitation. By incorporating community rehabilitation (physical therapy, exercise therapy, acupuncture and moxibustion, self-management education) in community care service, diverse service providers (rehabilitative professionals, social workers and grassroot government) are involved in this mode that can support people ageing in place. This enabled the cooperation between the rehabilitation department of our hospital, a 1000-bed tertiary hospital and the county government. Over one year of extensive consultation with representatives from 16 villages and 18 communities of the county, a novel community care service for older people was developed.

### Study design

A cross-sectional survey was conducted in Lishui county of Foshan during the period of January to March 2021. People who were 60 years of age and above and living in Lishui county for over six months were included in this study. We excluded those who had difficulty in communication due to serious physical illness or psychiatric disorder.

### Sample size determination

The sample size was determined on the assumed effect size of 0.50, referring the study of Liu, et al. [8]. To obtain reasonable estimates at 95% confidence level and 5% margin of error, a total sample size of 401 was needed, taking into account 5% non-response. This study included 413 senior residents allowing detection of significant differences with a power of 0.89 calculated by Gpower software.

### Sampling procedure

Lishui county is composed of 16 villages and 20 communities. Prior to the sampling procedure, we hosted a meeting with heads of villages/communities and representatives from social work organizations on October 10th, 2020. We first explained the aims and target population of this community care service to each attendee. Then the healthcare program was introduced including adopted therapies, frequency of rehabilitation services and follow-up visits. After addressing their questions, they were encouraged to spread the information. Brochures regarding the community care were also given to them. A staged cluster-sampling method was adopted to select participants. First, 8 villages and 10 communities were randomly selected. From each village or community, 23 residents were randomly selected from the roster that includes residents over 60 year old provided by the county government. Systematic random samplings were used to select villages/communities. Then computer-generated random numbers were used to determine specific residents. The procedure of randomization was conducted by an administrative person not involved in subject recruitment. The researchers were blinded in the process of recruitment. Participants were asked to sign a written consent form before the beginning of the interview. For those who had difficulty in writing, written informed consent was obtained from their spouses/children. The oral informed consent was recorded when the older people or their family members cannot write.

### Data collection

Data were collected using a structured questionnaire composed of three sections. The development of the questionnaire was based on Andersen- Nyman model of health services utilization [11]. After the pilot test on older people at community care centers, a manual was created to provide suggestions on how to ask questions so that older adults can understand. Eligible older people were contacted by the investigators with the help from the government staff. After explaining the research protocol and obtaining informed consent, participants were interviewed face-to-face at the community care centers. For those who preferred to be interviewed at their residence, we came to their homes. The data collection phase was completed with the help of 10 post-graduate students. They were trained for a day by the principal investigator covering research objectives, interview skills, quality control and research ethics. Approximately 15 minutes was spent on the total interview and each individual was reimbursed with a small gift after completion.

### Variables and measures

#### Dependent variables

The dependent variable was the willingness to receive community care for older people measuring by the question‘Would you be willing to receive community care service?‘with the option yes or no. The interviewer first explained this novel community care service, then the interviewer asked the respondent whether he or she understood the concept.

#### Independent variables

The independent variables were classified into three categories according to Andersen-Nyman model [11]. 1) Predisposing variables, such as age, gender, marital status, education and having children; 2) Enabling variables such as living arrangement, health insurance, household income and walking time from current residence to the nearest community care center; 3) Needing variables, such as physical activity over the past two weeks, number of non-infectious chronic diseases and activity of daily living (ADL) disability.

Physical activity was assessed by the International Physical Activity Long Volume (IPAQ-L). It covers four domains with a summary score which can be categorized into three levels (low, moderate and high). The validity of Chinese version has been confirmed [12]. The ADL disability was measured by Katz Index Scale. It includes six items and each item has two response choices. If any answer was ‘dependent’, the seniors were categorized as ADL disability. This scale had good reliability (Cronbach alpha>0.80) and validity (content validity index, CVI>0.90) to assess functional status of older people in China [13].

### Statistical analysis

The primary data was entered into Epidata 3.1, then analyzed by SPSS 20.0.Variables were presented as counts and proportions. Chi-square test was used to assess the differences in predisposing variables, enabling variables and needing variables between older people who were willing to receive community care service and those who were not. Multivariate regression analysis was used to identify factors that were significantly associated with willingness to receive community care for older people after controlling for possible confounders. Odds Ratios (ORs) with 95% confidence intervals (95% CIs) were calculated to measure the strength of association. Estimates with *p*-values less than 0.05 were considered statistically significant.

### Ethics

Ethics approval was obtained from the Research Ethics Board of Southern Medical University. All participants provided informed consent before being interviewed.

## Results

### Socio-demographic characteristics of study participants

Of the 413 older people approached to participate, 399 (96.6%) consented to participate in the survey. Among those who consented to participate in the survey, 396 (99.2%) completed the interviews. Of the 396 older people who completed the interviews, 245 (61.9%) were willing to receive community care.

Of all the respondents, the majority of older people were at the ages of 70 ~ 79 (46.5%), with more men (52.0%) than women (48.0%). More than three-quarters (80.3%) of the participants were married and a similar proportion (71.4%) had an education of primary school or below. 67.7% of the participants earned between 4000 and 7999 Chinese Yuan per month, followed by those who earned more than 8000 Chinese Yuan (32.3%) (Table 1).

**Table 1:** Socio-demographic features of participants

Variables	Frequency	Percentage (%)
Age		
60 ~ 69	169	42.7
70 ~ 79	184	46.5
80 ~ 91	43	10.8
Gender		
Male	206	52.0
Female	190	48.0
Marital status		
Married	318	80.3
Widowed/Never married	78	19.7
Education		
Primary or below	283	71.4
Junior middle school	67	17.0
Senior middle school or more	46	11.6
Annual monthly income (CNY)		
4000~	268	67.7
≥8000	128	32.3

*CNY* Chinese Yuan

### Willingness to receive community care for older people

The proportion that was willing to receive community care was significantly higher among older people who were living alone (83.2%, *n* = 153) than among those who were living with spouses (52.2%, *n* = 71) or children/others (27.6%, *n* = 21, *P* = 0.000). The proportion was also significantly greater among older people who lived closer to the community care center (walking time ≤ 15 minutes) than among those who lived far away (80.2%, *n* = 126 and 49.8%, *n* = 119 respectively, *P* = 0.000). In addition, the proportion of older people with ADL disability who were willing to receive community care was significantly higher than those without a disability (71.9%, *n* = 190 and 41.7%, *n* = 55 respectively; *P* = 0.000; Table 2). There were, however, no statistically significant variations in the proportions willing to receive community care by the other factors considered (Table 2).

**Table 2:** Willingness to receive community care among seniors

Variables Total	Willingness to receive community care	χ^**2**^	***P***
	Percentage (n)		
Predisposing factors		0.104	0.949
Age			
60 ~ 69,169	62.7(106)		
70 ~ 79,184	61.4(113)		
80 ~ 91 43	60.5(26)		
Gender		0.103	0.757
Male 206	62.6(129)		
Female 190	61.0(116)		
Marital status		0.004	1.000
Married 318	61.9(197)		
Widowed/ Never married 78	61.5(48)		
Education		0.040	0.980
Primary or below 283	61.8(175)		
Junior middle school 67	61.2(41)		
Senior middle school or more 46	63.0(29)		
Having children		0.066	0.772
Yes 384	61.9(238)		
No 12	58.3(7)		
Enabling factors			
**Living arrangement**		**78.475**	**0.000**
**Alone 184**	**83.2(153)**		
**With spouse 136**	**52.2(71)**		
**With children/others 76**	**27.6(21)**		
Health insurance		0.035	0.904
NCMS 301	62.1(187)		
MIUES 95	61.1(58)		
Annual monthly income (CNY)		0.014	0.993
4000 ~ 268	61.9(166)		
≥8000 128	61.7(79)		
**Walking time from current residence to the nearest community care center**		**37.270**	**0.000**
**≤15 minutes 157**	**80.2(126)**		
**>15 minutes 239**	**49.8(119)**		
Need factors			
Regular physical activity over the past two weeks		0.043	0.979
High 116	61.2(71)		
Medium 128	62.5(80)		
Low 152	61.8(94)		
Number of non-infectious chronic disease		0.116	0.944
0 38	63.1(24)		
1176	62.5(110)		
≥2182	61.0(111)		
**ADL disability**		**34.250**	**0.000**
**Yes 264**	**71.9(190)**		
**No 132**	**41.7(55)**		

*NCMS* New cooperative medical scheme, *MIUES* Medical insurance for urban employee scheme

Factors associated with willingness to receive community care.

Table 3 shows the factors associated with willingness to receive community care at the time of investigation. Seniors who were living alone or with spouses were about two times significantly more likely to receive community care compared to those who were living with children/others (OR = 2.19, 95%CI:2.44 ~ 3.19 and OR = 2.02, 95%CI:1.39 ~ 2.23, respectively). Similarly, older people who lived closer to the community care center were about two times significantly more likely to receive community care compared to those who lived far away (OR = 2.10, 95%CI:1.61 ~ 2.41). In relation to ADL disability, older people with ADL disability were three times significantly more likely to receive community care compared to those without a disability (OR = 3.13, 95% CI: 2.38 ~ 3.29).

**Table 3:** Odds ratios from multivariate logistic regression analysis examining factors associated with the willingness to receive community care service for older people in Foshan, China

Covariates	***OR***	***SE***	***95%CI***	***P***
Living arrangement				
Alone	2.91	0.15	2.40 ~ 3.19	**0.000**
With spouses	2.02	0.52	1.39 ~ 2.23	**0.005**
With children or others	–	–	–	–
Walking time from current residence to nearest community care center				
≤15 minutes	2.10	0.38	1.61 ~ 2.41	**0.000**
>15 minutes	–	–	–	–
ADL disability				
Yes	3.13	0.25	2.38 ~ 3.29	**0.000**
No	–	–	–	–

## Discussion

This cross-sectional study aimed to determine the proportion of older people who were willing to receive community care and analyze the influencing factors in a rural county piloting the incorporation of healthcare in community care service in Foshan, China. To our knowledge, this study was among one of the first studies that focused on a novel community care service for older people promoting community rehabilitation in rural China.

In the present study, 61.9% of the rural senior residents are willing to receive community care. This proportion is much higher than the 8.5% of a study conducted in three rural villages of south-central China [8]. Compared with a research on older adults’ attitudes towards institutional-based care offering integrated medical and social services in a community in southwest China, the proportion is also higher (11.9%) [14]. We believe positive attitudes towards community care in this study may result from public finance budget to support the system and specific healthcare (community rehabilitation). First, concerning the financing model, the present case had public finance budget and special government fund, which guaranteed the sustainable development of this care system. While in the case of south-central China, communities provided just minimal financial support to the older people who had no children [8]. In another case, older people were required to pay the management fees for access to this service [14]. The higher price may hinder them from choosing the care. Second, the present model embraced community rehabilitation through professional team both at care centers and at home. Although governments in the above-mentioned two cases encouraged service facility to conduct healthcare [8, 14], this service was mainly based on community health care, the foundation and entry point for the health system in China. Community physicians mostly treat uncomplicated conditions and have no long-term relationship or follow-up with any patients. There is no rehabilitation program for older people with chronic diseases. Because older people often face a long-term medical condition with lasting functional impairments, community rehabilitation plays an important role in improving health outcomes and reducing healthcare costs [14]. Rehabilitation involves preliminary assessment, goal-setting, intervention (i.e physical therapy, exercise therapy, acupuncture and moxibustion, self-management education) and reassessment. The active engagement of the older people, supported by professional team, could help fulfill the functional and psychosocial concerns needed to achieve better effects.

In the current study, the results revealed that living arrangements, walking time from current residence to the nearest community care center and ADL disability affected the willingness to receive community care. Older people in an empty-nest family (living alone or with spouse only) in our study were significantly more likely to receive community care compared with their counterparts living with children/others. This finding is consistent with another study that found healthy older people living in their own home alone are likely to accept community care [8]. This may be more in line with the norms of traditional Chinese Confucian culture. In rural areas, government-run care institutions target those older people who have no children, no income, and no relative. Indeed, virtually all residents in these rural institutions are welfare recipients, who are eligible for the guarantees, such as food, clothing, housing, medical care, and burial expenses. Therefore, community care is a good option for those who were living alone. On the other hand, due to the strong cultural norms of familial loyalty and collectivism, older people who lived with children preferred family care [8]. If they live in a care institution for older people or join community care, their children might be considered unfilial. The finding suggests that education campaigns should be launched to eliminate the stigma surrounding community care [8]. The governments should also increase awareness around community care to reduce the burden of family caregivers.

As an enabling factor, walking time was associated with higher likelihood of community care utilization willingness. Previous studies have shown that distance is an important determinant of health services usage [15]. There are two possible explanations for such an outcome. First, in rural areas, as young labor force migrated to cities, older people are rarely cared for by their children [4]. In this case, older adults prefer to live in a familiar environment and access the services provided within 15 minutes walking time. Second, many older people have to give up their idea of institutional care because of long distance and negative reputation. Because of these, governments in developed regions have set up care centers in the community as one of the important pillars of care for older people. Thus, the community plays a gatekeeper role in older people’s health and ensures that older adults live in a familiar environment and maintain China’s cultural norms. The finding indicates that site selection for future community care centers should consider geographic locations which can maximize convenience for senior residents.

Furthermore, the study demonstrated that seniors with ADL disability were more likely to receive community care. Essentially, some disabilities are strongly related to poor physical and psychological health, leading to high demand for continued rehabilitation [8]. Under this pattern community rehabilitation was adopted which seemed to be an appealing choice for older adults. The finding attaches importance to the specific healthcare when planning and offering community care for older people in rural areas.

It is worth noting that we did not find an association between willingness to receive community care and educational level, which was different from those research showing that older people with better education are open-minded, who will be more willing to accept the new pattern [8, 9, 14]. All significant results in our study were related to enabling factors and needing factors such as living arrangement, convenience and ADL disability. One possible explanation was that universal access to the community care service was offered and older people need not to pay extra fees.

This study has several limitations. First, a cross sectional study did not allow for establishing causal relationships between willingness to receive community care for older people and the factors associated with it. Second, information including economic status was self-reported, and participants might have provided responses they feel socially desirable. Third, while this study is among one of the first to examine willingness to receive a novel care service for older people among rural population in southern China, the findings may not be generalisable to other regions in China. Fourth, some components of community care, such as life assistance, fitness and cultural activities have not been explored, which are the future research directions. Further study is also needed to concentrate on how to set up a suitable long-term care system for older people in rural areas.

## Conclusions

The proportion of older adults who were willing to receive this novel community care service was comparatively high. Moreover, living alone, convenience and ADL disability were found to be important factors influencing willingness to receive community care among rural seniors. Government and social media should encourage and publicize the practice of community rehabilitation in community care agencies. Moreover, the findings suggest the need for policies and programs to not only improve the provision of this novel community care service but also promote its uptake among older people in the study setting.

## Data Availability

The datasets used and/or analyzed during the current study are available from the corresponding author on reasonable request.

## References

[1] Yang W, Wu B, Tan SY, Li BQ, Lou VWQ, Chen ZA (2021). Understanding health and social challenges for aging and long-term care in China. Res Aging.

[2] Haunch K, Thompson C, Arthur A, Edwards P, Edwards P, Goodman C, et. al. (2021). Understanding the staff behaviours that promote quality for older people living in long term care facilities: A realist review. Int J Nurs Stud.

[3] Zhang L, Yang J (2019). The different models of community eldercare service in China. Front Sociol.

[4] Wu L, Huang Z, Pan Z (2021). The spatiality and driving forces of population ageing in China. PLoS One.

[5] Ministry of Civil Affairs of the People’s Republic of China. The fourth sampling survey of the living conditions of the elderly in urban and rural areas in China. Beijing: China Statistics Press; 2016.

[6] Yeung WJ, Thang LL (2018). Long-term care for older adults in ASEAN plus three: the roles of family, community, and the state in addressing unmet eldercare needs. J Aging Health.

[7] Chen L, Ye M, Kahana E (2018). "Their today is our future": direct carers' work experience and formal caring relationships in a community-based eldercare program in Shanghai. J Appl Gerontol.

[8] Liu ZW, Yu Y, Fang L, Hu M, Zhou L, Xiao SY (2019). Willingness to receive institutional and community-based eldercare among the rural elderly in China. PLoS One.

[9] Xing Y, Pei R, Qu J, Wang J, Zhou H, Wang Z, et. al. (2018). Urban-rural differences in factors associated with willingness to receive eldercare among the elderly: a cross-sectional survey in China. BMJ Open.

[10] Wang Z, Xing Y, Yan W, Sun X, Zhang X, Huang S, et. al. (2020). Effects of individual, family and community factors on the willingness of institutional elder care: a cross-sectional survey of the elderly in China. BMJ Open.

[11] Zhou C, Ji C, Chu J, Medina A, Li C, Jiang S, et. al. (2015). Non-use of health care service among empty-nest elderly in Shandong, China: a cross-sectional study. BMC Health Serv Res.

[12] Zhou P, Hughes AK, Grady SC, Fang L (2018). Physical activity and chronic diseases among older people in a mid-size city in china: a longitudinal investigation of bipolar effects. BMC Public Health.

[13] Chen W, Fang Y, Mao F, Hao S, Chen J, Yuan M (2015). Assessment of disability among the elderly in Xiamen of China: a representative sample survey of 14,292 older adults. PLoS One.

[14] Dong X, Wong BO, Yang C, Zhang F, Xu F, Zhao L, Liu Y (2020). Factors associated with willingness to enter care homes for the elderly and pre-elderly in west of China. Medicine (Baltimore).

[15] Liu D, Meng H, Dobbs D, Conner KO, Hyer K, Li N, Ren X, Gao B (2017). Cross-sectional study of factors associated with community health centre use in a recently urbanised community in Chengdu, China. BMJ Open.

